# Geographical Parthenogenesis in Alpine and Arctic Plants

**DOI:** 10.3390/plants12040844

**Published:** 2023-02-13

**Authors:** Elvira Hörandl

**Affiliations:** Department of Systematics, Biodiversity and Evolution of Plants (with Herbarium), University of Goettingen, 37073 Göttingen, Germany; elvira.hoerandl@biologie.uni-goettingen.de

**Keywords:** apomixis, biogeography, DNA methylation, ecology, hybridization, polyploidy, stress response

## Abstract

The term “Geographical parthenogenesis” describes the phenomenon that asexual organisms usually occupy larger and more northern distribution areas than their sexual relatives, and tend to colonize previously glaciated areas. Several case studies on alpine and arctic plants confirm the geographical pattern, but the causal factors behind the phenomenon are still unclear. Research of the last decade in several plant families has shed light on the question and evaluated some of the classical evolutionary theories. Results confirmed, in general, that the advantages of uniparental reproduction enable apomictic plants to re-colonize faster in larger and more northern distribution areas. Associated factors like polyploidy seem to contribute mainly to the spatial separation of sexual and asexual cytotypes. Ecological studies suggest a better tolerance of apomicts to colder climates and temperate extremes, whereby epigenetic flexibility and phenotypic plasticity play an important role in occupying ecological niches under harsh conditions. Genotypic diversity appears to be of lesser importance for the distributional success of asexual plants. Classical evolutionary theories like a reduced pressure of biotic interactions in colder climates and hence an advantage to asexuals (Red Queen hypothesis) did not gain support from studies on plants. However, it is also still enigmatic why sexual outcrossing remains the predominant mode of reproduction also in alpine floras. Constraints for the origin of apomixis might play a role. Interestingly, some studies suggest an association of sexuality with abiotic stresses. Light stress in high elevations might explain why most alpine plants retain sexual reproduction despite other environmental factors that would favor apomixis. Directions for future research will be given.

## 1. Introduction

The term Geographical Parthenogenesis (GP) was originally introduced by Vandel (1928) [1] and described the phenomenon that sexual and asexual organisms have different geographical distributions. Later on, several authors described geographical parthenogenesis for animals and plants, and refined the pattern: asexual organisms tend to have larger distribution areas than their sexual relatives, and they colonize more frequently in higher latitudes, and in previously glaciated (or otherwise devastated) areas, whereby these features can occur alone or in combination [2,3,4,5,6,7]. Here I will update the known case studies, and discuss the causality with a focus on arctic and alpine plants of the Northern hemisphere, where biogeographical and climatic pre-conditions favor GP scenarios: firstly, these areas provide large landmasses in the circumarctic-circumboreal zone, secondly, several high mountain systems with elevations and vegetation above timberline exist, and thirdly, these areas were covered by ice-shields during cold periods of the Pleistocene, and left after postglacial glacier retreat huge areas for re-colonization. The climatic oscillations of the Pleistocene resulted in range fluctuations of plant and animal species, thus causing opportunities for secondary contact hybridization [8,9,10]. Hybridization, in turn, is regarded as the most important factor for the evolutionary origin of asexual lineages [11]. Most examples of arctic and alpine GP patterns are documented in Europe and North America [2,4,12]. An update of case studies indeed suggests that a majority of taxa or complexes (71%) do have great proportions of their geographical distributions in alpine and/or arctic regions (Supplementary Table S1). A GP pattern appeared also in functionally asexual plants reproducing via permanent translocation heterozygosity in the genus *Oenothera* [13]. GP patterns were observed also in vegetatively propagating plants (e.g., in *Allium*, [14]). However, also apomictic-sexual taxon pairs without typical GP patterns have been detected (e.g., *Hieracium intybaceum* in the European Alps [15], or *Boechera* species in North America [16]).

From its discovery onwards, geographical parthenogenesis has been enigmatic, and the causal factors have been under dispute [2,5,17]. Classical theories emphasize the advantages of asexuality per se, such as the advantage of uniparental reproduction for colonization as single individuals can act as founders of populations, a principle known as “Bakers’ law” [18]. Other authors stressed interactions of cytotypes and postulated introgression of asexuality into sexual populations, resulting in the reduction or extinction of the latter [19,20]. A set of ecological theories regard the interplay of niche occupation and the population genetic structure of asexuals: Asexual hybrids could have advantages as general-purpose genotypes (GPG) that can survive in several niches [21], or by producing broad arrays of clonal lineages that would better explore the resource space than sexual species (Frozen Niche Variation model, FNV, [22,23]). For plants, already previous authors stressed that the predominant polyploidy in asexual plants could have positive effects on asexuals and provide a better adaptivity to cold climatic conditions [2,5,24]. Biotic interactions were also thought to have indirect effects: asexual clones could perform better in colder climates where the pressure of parasites and pathogens would be expected to be lower than in warmer regions (Red Queen model, [17]). 

None of these classical theories exclusively explained the patterns, and hence some authors postulated combinations of factors [5]. Research of the last decade added several new case studies on GP occurrences or re-evaluated some older records of candidates of GP (Supplementary Table S1). Associations between main features as given in the literature will be discussed in the respective sections to shed some light on the “classical” hypotheses, like reproduction modes (Section 2), polyploidy (Section 3), and ecological factors (Section 4). However, recent research revealed that several other factors have to be considered: stress response, phenotypic plasticity, and epigenetic flexibility to acclimate to environmental conditions [25,26,27], see Section 5. Here I will provide an overview of the findings of the last decade to shed light on old and new questions about GP in plants. 

## 2. Factors Related to Mode of Reproduction

### 2.1. Uniparental Reproduction (Baker’s Law): Autonomous Apomixis Versus Pseudogamy

**Figure 1:**
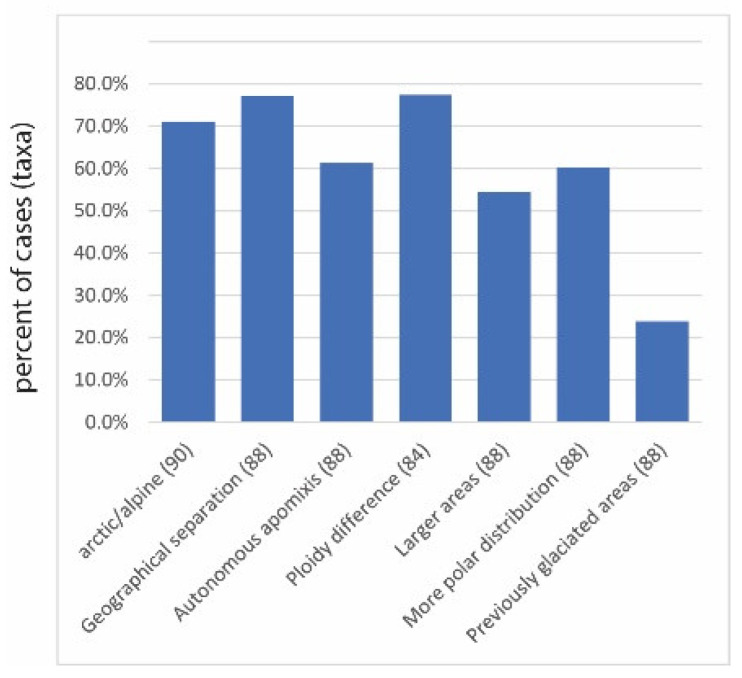
Percentages of taxa (no. of respective total cases in parentheses, based on presence/absence matrix of occurrences in Supplementary Table S1) with (at least partial) arctic/alpine distribution, geographical separation of apomicts and sexuals, autonomous apomixis, ploidy difference between sexuals and apomicts, larger areas of apomicts than sexuals, apomicts with a more polar (northern/southern) distribution than sexuals, and exclusive occurrence of apomicts in previously glaciated areas (see references in Table 1).

According to Baker’s law, uniparental reproduction is advantageous for colonization because a single individual can found a population, which applies both to selfing and apomixis [18]. Apomixis is in flowering plants defined as asexual reproduction via seeds [11] and occurs in many different developmental pathways (reviewed by [11]). Gametophytic apomixis involves the formation of an unreduced embryo sac out of a somatic nucellus cell (apospory) or an unreduced megaspore (diplospory); the egg cell develops parthenogenetically into an embryo. Sporophytic apomixis involved the development of embryos out of a nucellar or integumental cell. Seeds carry one (or more) embryo(s) that are clones of the mother plant. Both modes of reproduction can be associated with GP patterns, whereby gametophytic apomixis appears to be more frequent (Table 1). Different effectivity in colonization, however, can be assumed from the mode of pollination (pollen-dependent pseudogamy versus pollen-independent autonomous apomixis). Indeed, autonomous apomixis occurs in more than 60% of reported cases (Figure 1).

In most plant families apomictic seed formation is still dependent on pollination because sperm nuclei have to fertilize the polar nuclei for proper development of the endosperm, the nutritious tissue for the embryo (pseudogamy). Most plants are hermaphrodites and can use their own pollen for this purpose [71,77,78,79,80,81,82], and hence they are not only independent from another individual as pollen donor, but also are largely independent of a vector carrying pollen (wind or animals). This independence from pollen transfer and another mating partner gives a double advantage to colonization, as a single individual can found a population [5,18]. This advantage appears to overcome even cases of lower fertility of apomictic plants compared to sexuals, as observed in the alpine herb *Ranunculus kuepferi* with GP in the European Alps [66,67]. A simulation study considering modes of reproduction, fertility, ecology, and colonization history of this species revealed that the apomicts clearly showed a much more efficient postglacial recolonization of the previously glaciated areas of the European Alps despite a lower seed set [83].

A potential disadvantage of self-pollination and pseudogamy could be caused by herkogamy within flowers, ie. pollen would not come into contact with the stigmas, or dichogamy, ie. pollen maturation would be asynchronous with that of the stigma. In these cases, pollinators would be still required for successful pseudogamy, because insects moving around within the flower would disperse the pollen, and pollinator visits between flowers in different developmental stages within the same individual would overcome dichogamy. No detailed case studies addressed this question so far for pseudogamy (one case study on breeding systems of *Aechmea* revealed autonomous apomixis [84]). A second major constraint of pseudogamy is potential endosperm imbalance after fertilization of the polar nuclei, i.e., deviations from the optimal 2:1 ratio of maternal to paternal genome copies in the endosperm (e.g., [85]). Pseudogamous plants show a great variation in maternal:paternal genome contributions in the endosperm [66,85,86,87,88], and endosperm imbalance can result in a lower seed set [85]. Hence, although pseudogamy does allow for uniparental reproduction, it is constrained by potential pollinator limitation and/or by potentially reduced female fitness. 

In Asteraceae, many species reproduce via autonomous apomixis, which means that pollination and fertilization of polar nuclei are not necessary for proper seed formation [4,89]. Asteraceae are strongly represented in the cases of GP (see Table 1) which supports the hypothesis that uniparental reproduction without any need for pollen is an important advantage to asexuality [4]. Endosperm imbalance is no more an issue as other tissues take over the nutritious function of the embryo. Some plant families like orchids do not form endosperm at all. Under these considerations, autonomous apomixis is expected to be more efficient for GP. Indeed, autonomous apomixis correlates significantly with larger distribution areas, and with more polar (northern/southern) distribution patterns (Supplementary Table S2). Pollen-independent apomixis appears to be an advantage to expand spatial distributions. 

However, there was no significant correlation between autonomous apomixis to previously glaciated areas. There might be too few case studies to reveal an association. *Hieracium pilosella* is a widespread species with a GP pattern over the whole of Europe [37]. In a study comparing both sexual and apomictic cytotypes on glacier fore fields in the Swiss Alps, apomixis was more frequent in odd-number ploidy levels, and these occurred closer to the glacier snout than other cytotypes [90]. The authors concluded that apomixis provided reproductive assurance in odd-ploid cytotypes by avoiding meiosis disturbances and loss of fertility, and hence a colonization advantage in the most recent ice-free areas. Reproductive assurance without pollination was further confirmed in *Hieracium alpinum,* a widespread herb distributed in the European mountain system and in Northern Europe. Diploids are self-sterile and reproduce sexually, whereas triploids are autonomous (pollen-independent) obligate apomicts [38]; female fertility does not differ under natural conditions. Only triploids colonized previously glaciated areas in the Alps and in Scandinavia, whereas diploids occur in the Carpathians. The GP pattern was referred to as a better colonization ability and a more stable reproductive system of apomicts [38]. In sympatrically cultivated apomictic and sexual populations, apomicts had much higher fertility, which was explained by mate limitation acting for sexuals, but not for apomicts [91]. However, colonization history may influence GP patterns. In *Hieracium intybaceum*, diploids appeared to be sexual outcrossers, whereas tetraploids are autonomously apomictic. The diploids colonized most parts of the Alps whereas apomicts were confined to a small, disjunct area in the Western parts of the Alps, the Vosges and Schwarzwald mts. [15]. The authors explain this untypical pattern which only involves geographical separation of cytotypes (sensu [1]) with a very young evolutionary origin of apomicts [15].

Nevertheless, uniparental reproduction is also possible for sexual plants via selfing, and this taxonomically widespread trait reduces the advantage of asexuality over sexuality [5,92]. For instance, regular selfing of sexual plants may hinder the evolution of GP in the genus *Boechera* (Brassicaceae), which comprises both sexual regular selfers and apomictic species in North America [87]. But, only a few species show some geographical separation (Table 1). A further peculiarity of *Boechera* is the occurrence of apomixis on the diploid level [93], which means that positive side effects of polyploidy for apomicts would disappear [16]. The almost lack of the GP pattern in this genus might be due to a combinational effect as well as its presence in other genera. 

Taken together, all studies so far confirmed the advantage of uniparental reproduction of asexuals for colonization. The effect is apparently stronger in autonomous than in pseudogamous apomicts, and probably stronger in the case of self-sterility of sexual relatives. However, uniparental reproduction alone is not the only causal factor as some apomictic species do not exhibit GP despite the respective modes of reproduction. 

### 2.2. Reproductive Interactions of Cytotypes

Sexual reproduction is in angiosperms the ancestral state [94], and apomixis arises in sexual populations [11]. This means that at least initially sexual and apomictic individuals coexist and can interact with each other. In a mixed population of diploid sexuals and polyploid apomicts, apomictic polyploid individuals can overcome minority cytotype disadvantages [95] better than sexual polyploids, because they would not or only to a minor extent be fertilized by the pollen of the surrounding diploid sexuals. With apomixis, even a single plant can produce clonal offspring with the maternal ploidy level. This factor was also seen as a major advantage of GP for establishing polyploid apomictic populations in the alpine herb *Ranunculus kuepferi* [83]. 

On the other hand, pseudogamous apomicts usually maintain functional pollen, and hence can fertilize surrounding sexual individuals without being fertilized themselves by diploids. This unidirectional hybridization would theoretically result in the introgression of apomixis into sexuals and hence the extinction of sexuality [19,96]. However, experimental crossings in *Ranunculus auricomus* revealed that pollinations of diploid sexuals with pollen from tetraploid apomicts results in a very low seed set, probably because the different ploidy levels and also induced selfing of sexuals (Mentor effect) acted as efficient crossing barriers [97]. At higher ploidy levels, however, these crossing barriers between cytotypes appear to be less efficient. The crossability of polyploid cytotypes was studied in *Potentilla puberula*, a widely distributed species in the European Alps with tetraploid sexuals and higher polyploid apomictic cytotypes (5x, 6x, 7x, 8x, and 9x) [98,99]. In experimental heteroploid crosses, the seed set was reduced in sexuals, but not in apomicts, compared to homoploid crosses. This asymmetrical interference gives an advantage to apomicts [99]. The sexuals and apomicts in *Potentilla* show a sympatric distribution in the Alps, but with differentiation of reproduction modes along altitude and habitats [74]. Because of sympatry, interactions of cytotypes influence distribution patterns in natural populations, but ecological differentiation plays a role as well [74,100]. 

Pollen fertility is usually much reduced in autonomous apomicts [77], but nevertheless, remnant viable pollen can still cause asymmetric interference in mixed populations [101]. Simulations of local reproductive interference in sympatric sexual and apomictic *Crepis* confirmed that sexuals occur mostly in spatial isolation, whereas apomictic taxa can co-occur [102]. Hence, the presence of apomicts can lead to local exclusion of sexuals and drive the spatial separation of reproductive types in the initial stages. However, most extant cases already do have spatially separated sexual/apomictic populations, so the effects of interactions are probably only initially important (Table 1). 

## 3. Polyploidy and Hybridization

Polyploidy was traditionally seen as a major factor fostering GP patterns [2,5,24]. However, its role as the only causal factor was questioned by the lack of general patterns of geographical polyploidy in the many sexual plants ([5]). In the updated review (Table 1), a significant correlation appeared only for Vandel’s [1] basic pattern that diploid sexuals and polyploid apomicts have separated distributions (Supplementary Table S2). However, there was no significant association of polyploid apomicts with any of the other GP features (larger distributions, more polar distributions, or previously glaciated areas; Supplementary Table S2). The association with autonomous apomixis was also not significant. However, combinational effects were postulated [5], and actually confirmed in a simulation study on *R. kuepferi* on postglacial colonization of the Alps [83].

The indirect role of polyploidy for establishing separate distributions of apomicts and sexuals could be manifold. First, during initial sympatry, diploid sexuals might disappear in mixed populations because of a unilateral minority cytotype disadvantage (see Section 3). Second, polyploidy could be connected with a shift to self-fertility, which is an advantage for pseudogamous apomicts (see Section 2). Due to the bypass of meiosis, apomicts avoid loss of heterozygosity and inbreeding depression in the offspring, which otherwise reduces the fitness of selfing diploid populations. Inbreeding in small, diploid self-incompatible populations results in lower fitness and is disadvantageous to diploid sexual progenitors [76,103,104,105]. Third, polyploids, specifically allopolyploids, have higher levels of heterozygosity, both on the population level [106], but also as intragenomic heterozygosity ([65]). However, GP is also found in case studies with the same heterozygosity levels of sexuals and apomicts (e.g., in the autopolyploids *Antennaria friesiana* [29], and *R. kuepferi* [66,80,107], but also in allopolyploids of *Crataegus* [72,82]). Heterozygosity could help polyploids to buffer deleterious mutations and tolerate more extreme climatic conditions ([65]), or enable them to adapt to novel niches (see Section 5). Specifically, a recent global study on distributions of polyploid plants (sexual and apomictic) found a significant correlation between polyploids to colder climates [108]. 

Previous authors emphasized also the influence of hybridity on GP patterns, mainly based on examples from animals [3]. However, for plants, this correlation is less straightforward, as not only allopolyploids, but also many autopolyploids are known to exhibit GP (e.g., *Antennaria friesiana*, *Ranunculus kuepferi*, *Hieracium alpinum, Potentilla pusilla*). Moreover, most *Boechera* apomicts are diploid hybrids [49] but do not show typical GP patterns (Table 1). Altogether the information on the evolutionary origin of apomictic polyploids is still too incomplete for a more detailed evaluation of the influence of hybridity on GP patterns. 

Both polyploidy and hybridization are regarded as the major evolutionary pathways for the origins of apomixis in natural populations [11]. However, successful natural origins are probably rare, as various genetic and epigenetic changes of regulatory mechanisms have to be combined for the establishment of functional apomictic seed formation, and there is no universal pathway to apomixis [109,110,111,112,113]. The rarity of the evolutionary origins of apomictic lineages may also constrain the frequency and expression of GP patterns.

## 4. Ecology

### 4.1. FNV and GPG: Does Genetic Diversity Really Matter?

Two classical models, frozen niche variation (FNV) and general-purpose genotypes (GPG), both rely on the assumption of clonality. In the first case, broad arrays of clones would occupy a larger resource space, while in the second scenario, single widely distributed clones would cover the larger resource space. Already previous literature surveys [7,64,106] demonstrated that the assumption of clonality is often not met because facultative sexuality and high genotypic diversity are widespread in apomictic plants. A “little bit of sex” is for plants probably under positive selection as even low frequencies of recombination can efficiently counteract the accumulation of deleterious mutations, even in polyploid genomes [114]. More recent population genetic surveys confirmed that GP is usually not associated with clonality of apomicts [16,51,65,80,100,115]. In such cases, the FNV and GPG models are simply not applicable. In *Boechera* species, range size is not correlated to allelic diversity and heterozygosity within populations [116]. Widespread generalist clones, however, were found in North American *Crataegus* [82]. In the alpine orchid genus *Nigritella*, high clonal diversity was found, but only with populations in the vicinity sharing the same genotypes [54]. In general, the influence of genotypic diversity or clonality on GP patterns appears to be less relevant for plants than predicted by classical models. 

### 4.2. Niche Shifts of Cytotypes

In the last decade niche modeling based on climatic data from WordCLIM or other databases became a novel and widely used method in evolutionary and ecological research (e.g., [108,117]). Several studies on GP applied these methods and revealed a general tendency to climatic niche shifts of apomictic polyploids. That is, no single clones, but rather the sexual vs. apomictic cytotypes as a whole exhibit niche divergence. A tendency to colder climatic conditions was observed in alpine *R. kuepferi* [67,118], in the widespread common dandelion *Taraxacum officinale* [44], in *Crataegus suksdorfii* widespread in North America [82], in boreal *Boechera stricta* in North America [49], in the coastal *Limonium ovalifolium/binervosum* complex in Europe [57], and in south American *Paspalum intermedium* [61]. This trait would fit the general pattern that polyploids globally occur more frequently in colder climates [108], and to the frequent trend of asexuals towards more polar distributions (see above). However, this trait to colder climates is not universal. In *Potentilla puberula,* asexuals tended to wetter and to more anthropogenic habitats, and niche divergence contributed significantly to the small-scale differentiation patterns of sexuals and asexuals [74,100]. In the first survey of apomicts in the Himalayan flora, apomicts occurred in habitats with higher soil moisture [119]. In *Ranunculus auricomus*, the distribution of apomicts tended to regions with lower light intensity [65]. Niche expansion of asexuals was observed in *Taraxacum* Sect. *Erythrosperma* [44], and in *Crataegus douglasii* [82]. Alien sexual and apomictic plants do not differ in their niche spectra [120].

In general, the data support the hypothesis that ecological niche shifts of apomictic cytotypes are associated with GP patterns (whereby the data from the older literature are too incomplete to perform statistical tests for all cases). However, there is not a uniform trend to a certain niche, and also different patterns appear in different taxa of the same genus (e.g., in *Ranunculus* and *Taraxacum*). Little is known about the physiological background of these niche divergence patterns (see Section 5) Understanding ecological niche divergence needs more case-wise studies on the most relevant abiotic factors. 

### 4.3. Do Apomicts Tend to Higher Elevations?

The classical reviews proposed a correlation of GP not only to higher latitudes but also to higher elevations [2,5], which would be also expected from the respective temperature gradients in mountains. However, the older literature did not provide detailed information on elevations for statistical tests and also the classical survey by Bierzychudek [2] discussed only one genus (*Townsendia*) as an example. In Central Europe, a survey of vegetation data suggested a correlation of apomicts to higher altitudes as the only ecological trait [6]. However, this study was based on a taxonomic assignment of apomixis and not on a site-per-site assessment of apomixis, and hence did not regard the infraspecific differentiation patterns of GP. Detailed examination of mode of reproduction per site (usually via flow cytometric seed screening; [121]) rather gives an equivocal picture: a study of all Asteraceae species of the European Alps (229 taxa) did not reveal a correlation of apomixis to higher elevations [122]. Likewise, in subnivale plants of the European Alps, representing the angiosperm flora at the highest elevations, only one of 12 species expressed apomixis [123]. On the other hand, *R. kuepferi* and *H. alpinum* showed clear correlations of apomixis to higher elevations in the alpine zone [38,67], but only for populations within temperate European mountains. A study on *Potentilla puberula* in the Alps, however, did not reveal differences in elevation between sexuals and apomicts [74]. The first detailed investigation of apomixis in the Himalayas in the Ladakh region revealed that apomixis is not correlated to elevation [119]. 

A problem with elevation is that comparisons only make sense within a mountain system and within the same latitudinal zone (e.g., Alps, Himalayas), because, with higher latitudes, the respective alpine vegetational belts move down to low elevations [124]. Therefore, for taxa with a large latitudinal range expansion, differences in elevation in the South disappear towards the North. For instance, apomicts of *Hieracium alpinum* occur in mountains of Central Europe between 1080–2570 m a.s.l. (mean 2020 m), the diploid sexual cytotypes between 1380–2120 m (mean 1777 m). The apomicts in Northern Europe grow between 200–1280 m (mean 738 m) (data from [38]). For complexes with a large north-south distribution, this effect might eliminate all elevational differences. Likewise, in *Amelanchier* widespread in North America, no correlation of apomixis to elevation was found, only to latitude [69]. In the *Ranunculus auricomus* complex, ranging from the Mediterranean to the Arctic of Europe, there was even a reverse pattern that sexuals occurred in higher elevations in Mediterranean mountains [65]. In altitudinal transects of *Taraxacum* sect. *Ruderalia* in Switzerland, sexuals were found in higher elevations [125,126,127]. Also in the South American cerrados, sexuals of the genus *Eriotheca* occur at higher elevations [53]. Such cases also fit global surveys of polyploid plants that did not reveal a clear correlation between polyploidy and elevation [108].

Within mountain systems, the actual ecological conditions depend not only on elevation but also on the microhabitat, e.g., north/south exposition, soil conditions, duration of snow layer, human influence, etc. Other effects of high elevation, e.g., high UV solar irradiation, or lower atmosphere pressure [124], are largely unexplored for apomictic plants. So far, detailed investigations within mountain systems are needed to get a full understanding of the effects of elevation and the associated ecological trends. 

### 4.4. Do Apomicts Tend More to Previously Glaciated Areas Than Sexuals?

Previous reviews also postulated that apomicts would colonize previously devastated areas such as glaciated or arid areas [2,3,5]. For plants, this means mostly re-colonization of areas that were covered by ice shields during cold periods of the Pleistocene. However, evaluation of pairs with apomicts only in glaciated areas and sexuals not, only about 24% of cases had such exclusive patterns, and the association to GP is statistically not significant (Supplementary Table S2). This is simply due to the observation that sexual relatives often occur in previously glaciated areas as well [2]. Only a few detailed phylogeographic studies are available but suggest that biogeographical history depends also on mountain topography. In *Ranunculus kuepferi* in the European Alps, only apomicts managed to overcome a general topographical barrier in the southwestern Alps, mostly by a niche shift to colder conditions, but not by faster spread [83]. The study on *Potentilla puberula* in European also found a stronger influence of niche shifts than of biogeographical history [100]. An interesting case is *Townsendia hookeri* in western North America, where sexuals were found in unglaciated refugia north and south of the previously glaciated areas, where only apomicts occur [47]. Glaciations probably caused vicariance of sexuals, whereas postglacial recolonization of the Central parts of the western North American mountain chains was conducted only by apomicts [47]. This study also underlines that the topography of mountain systems and locations of ice-free Pleistocene refugia influence the realized GP patterns.

### 4.5. Biotic Interactions: No Support for the Red Queen Model

The so-called Red Queen hypothesis postulates that sexuals have an advantage in areas/situations with high frequencies of biotic interactions because they produce genetically variable offspring that could respond better to co-evolving parasites and pathogen infections in a co-evolutionary arms race [128,129,130]. Accordingly, asexuals should have an advantage in areas with low biotic interactions, as it is regarded as typical for higher latitudes and altitudes. Few studies on plants with GP are available on this aspect and they would not support the theory [5]. An experimental study on diploid and polyploid *Parthenium argentatum* with respect to their tolerance to the pathogenic fungus *Verticillium dahliae* did not reveal differences between cytotypes [131]. Hartman et al. [17] analyzed the GP pattern of *Hieracium alpinum* in natural populations concerning the intensity and frequency of seed predators and found no significant differences between sexuals and apomicts, and no latitudinal gradients, despite a higher genotypic diversity of the sexuals. Seed predation per population was even positively associated with intra-population diversity. In a study on common dandelion populations (*Taraxacum* sect. *Ruderalia*) over a large latitudinal gradient, northern populations showed reduced infections of a seed-eating weevil species compared to southern populations, but no difference appeared in pathogenic rust fungi and pathogenic soil microbes [132]. But it remained unclear whether the patterns can be attributed to a less efficient response of asexuals in plants to their predators or pathogens.

The problem with the Red Queen is that in plants many basic assumptions of the model are not met. First, clonality is not a general feature of apomictic plants due to facultative sexuality and some diversity of genotypes (see Section 4.1). Second, the assumption of a weaker defense of asexual plants to herbivory or other biotic attacks in higher latitudes is simply not supported by empirical data [133,134]. Large-scale surveys of chemical compounds rather suggest a greater resistance to herbivory of high-latitude plant species than of tropical species [134]. There is no difference in physical defenses [133]. Third, in biotic interactions, polyploidy of organisms could infer even better resistance or tolerance to biotic stressors, and this has been shown for plants and animals [135]. For plants, polyploidy alters profiles of chemical compounds and hence defense against biotic attacks [136]. So far, polyploidy appears to be an important indirect player in biotic interaction scenarios. Therefore, the Red Queen model is probably too simplistic or even inapplicable to explain GP in plants. 

## 5. Physiology: Phenotypic Plasticity and Epigenetic Response

### 5.1. Cold Stress

The frequent occurrence of apomixis towards polar regions, and the general tendency of polyploids to colder climates [108] could have different physiological backgrounds: first, apomictic plants are more cold-tolerant because of polyploidy, and second, cold temperatures could directly induce apomictic reproduction [137]. These hypotheses are not mutually exclusive. Cold adaptation is in plants strongly under epigenetic control (see Section 5.3). The induction of apomixis by temperature extremes is most directly related to apomictic development. Low-temperature shocks are known to trigger unreduced gamete formation in plants by disturbing spindle formation at meiosis [138]. Although these mechanisms are better studied for male development, female development would be similarly affected resulting in unreduced megaspores (the diplosporous pathway) or failure of meiosis and somatic development (apospory). In experimental cold treatments in climate growth chambers, diploid alpine *R. kuepferi* produced few but significantly more apomictic seeds than in the warm-treated control, whereas the tetraploid apomicts remained unaffected [137]. Seed screening of diploid populations from field samples revealed a female triploid bridge with unreduced egg cell formation, which could be regarded as the first step for the establishment of apomixis in natural populations [139]. 

However, the reproductive development of apomictic plants remains to be studied. Many alpine plants perform flower preformation in the previous or even pre-previous summer season, whereby the timing of the critical phase of meiosis and gametogenesis can vary from species to species [124]. Flower preformation during the warm season could avoid the cold shocks during meiosis that would cause unreduced gamete formation. Flower preformation, but also higher freezing tolerance, would mean that plants occurring in the extreme subnival zones are so well adapted to low-temperature conditions that developmental pathways in flower buds would not be affected [123]. This adaptation could explain why apomixis is rare in plants of the highest elevations of the Alps. Under these assumptions, rather plants adapted to lower altitudes and moving upwards in re-colonization processes would experience cold shocks and express cold-induced apomixis. This hypothesis still needs to be tested. 

### 5.2. Light Stress

Light stress is specifically of interest for mountain plants because solar radiation (annual means) increases with altitude, and also the fraction of UV light increases with high altitude [124]. Moreover, alpine plants above the tree line and also above the dwarf shrub zone grow often in completely unshaded conditions. Many typical alpine plants are well adapted to solar radiation and even to high UV light exposure. The influence of light stress on the mode of reproduction is not well known. It has been shown experimentally in facultative apomictic lowland plants of the *Ranunculus auricomus* complex that light stress during the flowering bud stage can increase frequencies of sexual ovule development [88,140,141,142]. Photosynthetic stress in the reproductive tissues can result in oxidative stress within ovules which would initiate meiosis and sexual development [143,144]. Also, other kinds of oxidative stress (drought, starvation, hydrogen peroxide treatments) can trigger sexual reproduction in facultative apomictic plants [145,146]. In the widespread *Ranunculus auricomus* complex, the GP pattern showed a significant correlation of sexual seed formation to higher light intensities, which is due to the occurrence of sexual species in southern mountain systems at higher elevations than most apomictic taxa [65]. Experimental light treatments showed that polyploidy reduces the effect of light stress on sexual ovule formation [141], which might be due to a better quenching of excess light [141,144]. In *Hieracium alpinum*, lower foliar nitrogen and carbon content was found in triploid apomicts of Scandinavia compared to diploids in the Carpathians, which was related to lower solar radiation and lower precipitation in the Northern areas [147]. 

These observations could also explain why the association of apomixis to elevation is equivocal (see Section 4.3): higher solar radiation and stronger UV light exposure of high alpine plants would rather cause oxidative stress in reproductive tissues and stimulate the maintenance of obligate sexuality [123]. 

### 5.3. Epigenetic Control Mechanisms 

In plants, response to biotic and abiotic stresses is strongly modulated by epigenetic control mechanisms that influence gene expression patterns [148,149,150]. The best-known epigenetic mechanism is DNA methylation which can be involved in direct phenotypic response to abiotic and biotic stresses as well as in heritable changes of regulatory mechanisms [25,149,150,151,152,153]. The stress memory kept via methylation changes allows plants to rapidly acclimate and adapt to changed environments [150,153,154]. DNA-methylation changes are important for phenotypic plasticity and adaptation of evolutionarily young, apomictic lineages, despite low genetic divergence and variability [25]. Moreover, asexual reproduction would bypass the epigenetic resetting mechanisms during meiosis and gametogenesis, and hence could enhance the transgenerational transmission of epialleles [25,154]. DNA methylations can further silence transposable elements (TEs), which make up large portions of plants’ genomes; hence, asexual genome evolution might be strongly affected by TE dynamics [155]. In dandelions, heritable methylation changes in apomictic plant lineages could explain the high divergence of methylations and accession-specific TE profiles [156]. TE profiles have a strong influence on heritable gene expression patterns, some of which could reflect the functional divergence of lineages [157].

Epigenetic research has always focused on model organisms and crops, and few studies are available on wild apomictic plants in the context of ecological adaptations. The most comprehensive studies related to GP are available on alpine *R. kuepferi* by using methylation-sensitive AFLPs (MSAPs). Wild populations differed in their MSAP profiles between diploid sexual and tetraploid apomictic cytotypes, but also between obligate and facultative apomictic tetraploids [158]. Controlled temperature experiments in climate growth chambers revealed a significant loss of methylations after temperature changes in both cytotypes and also influenced growth [159,160]; gene expression analysis further confirmed a stress response after cold treatments, which was stronger in diploids than in tetraploids [161]. All results confirmed a better cold acclimation of tetraploids compared to diploids as a major factor for the GP pattern in this species [161].

Another model system is the common dandelion (*Taraxacum* sect. *Ruderalia*). Experimental abiotic stress treatments of apomictic clones resulted in partly heritable methylation changes as measured with MSAP profiles [162]. A study of methylations over a large geographical scale from Central Europe to Scandinavia revealed a strong dependence of epigenetic variation on the genetic background, but also a small fraction of sequence-independent epigenetic variation that may contribute to phenotypic variation and local adaptation [163]. A more detailed study on the heritability of stress-induced methylation changes revealed that transgenerational modification of methylation patterns is genotype and context-dependent, and mostly undirected [164]. In wild populations of dandelions, differences in flowering time are associated with methylation patterns but not with geographical distance [165]. 

A limitation of MSAP-based studies was the low number of markers and the lack of sequence-based information for the anonymous MSAP fragments which strongly limits functional information of methylation patterns. The resources needed for bisulfite-based sequencing methods to analyze complete methylomes were so far not feasible for non-model organisms, and for the number of samples that are required for biogeographical studies. However, detailed experimental work on apomictic plants testing acclimation/adaptation to key environmental factors compared to epigenetic profiling will be promising to understand the ecophysiological background of GP. 

## 6. Summary and Outlook

Research of the last decade has confirmed that GP is a widespread phenomenon for sexual-apomictic plant complexes and has shed light on general aspects of its causality. Most patterns show a geographical separation and range expansion of apomicts, while associations of GP to elevation and previous glaciations are rather equivocal, probably due to the topographical and climatic complexity of mountain systems. While the advantages of uniparental reproduction for apomicts are largely confirmed, the role of polyploidy seems to be an indirect one and is probably connected to the frequently observed climatic niche shifts of asexual polyploid cytotypes compared to their sexual progenitors. The background of these niche shifts might be rather a phenotypic plasticity and short-term adaptation of asexual lineages to altered abiotic conditions, probably driven by epigenetic variability rather than by genetic changes. 

Still, only a few model systems are comprehensively explored for these aspects, and further information on reproductive data, ecological, experimental, and molecular work is needed on many genera to confirm the here proposed traits. A major challenge is still to perform large-scale screenings of a representative sampling from large geographical areas, often involving complex mountain systems, and also high arctic regions that are not easily accessible and therefore not well explored. More detailed studies on alpine plants in different mountain systems of the world are wanted to understand the uncertain relations of apomixis to higher elevations and the specific physiological adaptations to extreme conditions at high altitudes. A broad application of the flow cytometric seed screening method is most promising for this task. However, for understanding the causality of the pattern, ecological and physiological studies, experimental work, and genetic/epigenetic studies are wanted. 

## Figures and Tables

**Table 1:** Overview of published studies on GP in plants. Arctic/alpine means position of the whole taxon or group; GA = gametophytic apomixis, SA = sporophytic apomixis; A = autonomous apomixis (pollen-independent), P = pseudogamous apomixis (pollen-dependent); S = any geographical separation of sexuals/apomicts; L = apomicts have larger distribution areas than sexuals; N = apomicts have a more polar distribution than sexuals (towards poles in the northern or southern hemisphere, respectively); G = only apomicts occur in previously glaciated areas, sexuals not. Associations between the main factors (Table 1) were calculated using Past vs. 4.03 [28] (see presence/absence matrix and details in Supplementary Table S1).

Taxon	Family	Arctic/Alpine	Type of Apomixis	Type of Pollination *	Ploidy Difference of Apomicts/Sexuals	GP Pattern **	References
*Antennaria friesiana*	Asteraceae	yes	GA	A	yes	S, L, N	[29]
*Antennaria monocephala*	Asteraceae	yes	GA	A	yes	S, L, N	[29]
*Antennaria parlinii*	Asteraceae	no	GA	A	no	S, G	[30]
*Antennaria rosea s.l.*	Asteraceae	yes	GA	A	yes	S, L, N	[31,32]
*Arnica alpina*	Asteraceae	yes	GA	A	yes	S, L, N, G	[33]
*Arnica amplexicaulis*	Asteraceae	yes	GA	A	yes	S, L, N	[33]
*Arnica angustifolia*	Asteraceae	yes	GA	A	yes	S, G	[33]
*Arnica chamissonis*	Asteraceae	yes	GA	A	yes	S, L, N	[33]
*Arnica lessingii*	Asteraceae	yes	GA	A	yes	S, L	[2]
*Arnica lonchophylla*	Asteraceae	yes	GA	A	yes	S, L, N, G	[33]
*Arnica louiseana*	Asteraceae	yes	GA	A	yes	S, L, N	[33]
*Arnica mollis*	Asteraceae	yes	GA	A	yes	S, L, N	[33]
*Chondrilla juncea*	Asteraceae	no	GA	A	yes	S, L	[7]
*Crepis acuminata*	Asteraceae	no	GA	A	yes	no	[2,34]
*Crepis bakeri*	Asteraceae	yes	GA	A	yes	S, L, N	[2]
*Crepis exilis*	Asteraceae	yes	GA	A	yes	S, L, N	[2]
*Crepis modocensis*	Asteraceae	yes	GA	A	yes	S, L, N, G	[2]
*Crepis monticola*	Asteraceae	yes	GA	A	yes	S, L, N	[2]
*Crepis occidentalis*	Asteraceae	yes	GA	A	yes	S, L, N, G	[2]
*Crepis pleurocarpa*	Asteraceae	yes	GA	A	yes	S, L, N	[2]
*Erigeron strigosus*	Asteraceae	no	GA	A	yes	S, L, N	[35]
*Eupatorium altissimum*	Asteraceae	no	GA	A	yes	S, L, N, G	[36]
*Eupatorium cuneifolium*	Asteraceae	no	GA	A	yes	S, L, N,	[36]
*Eupatorium lecheaefolium*	Asteraceae	no	GA	A	yes	S, L, N	[36]
*Eupatorium leucolepis*	Asteraceae	no	GA	A	yes	S, L, N	[36]
*Eupatorium pilosum*	Asteraceae	no	GA	A	yes	S, L, N	[36]
*Eupatorium rotundifolium*	Asteraceae	no	GA	A	yes	S, L, N	[36]
*Eupatorium sessilifolium*	Asteraceae	no	GA	A	yes	S, L, N, G	[36]
*Hieracium alpinum*	Asteraceae	yes	GA	A	yes	S, L, N, G	[37,38]
*Hieracium intybaceum*	Asteraceae	yes	GA	A	yes	S	[15]
*Hieracium pilosella* s.l.	Asteraceae	yes	GA	A	yes	S, N	[2,39,40]
*Parthenium argentatum*	Asteraceae	no	GA	P	yes	S, L, N	[41,42]
*Taraxacum Sect. Alpestria*	Asteraceae	yes	GA	A	yes	S, L, N, G	[43]
*Taraxacum Sect. Erythrosperma*	Asteraceae	yes	GA	A	yes	S, L, N	[44]
*Taraxacum Sect. Ruderalia*	Asteraceae	yes	GA	A	yes	S, L, N	[7,44]
*Townsendia condensata*	Asteraceae	yes	GA	A	yes	S, L, N, G	[45]
*Townsendia exscapa*	Asteraceae	yes	GA	A	yes	S, L, N, G	[45]
*Townsendia grandiflora*	Asteraceae	yes	GA	A	yes	no	[45]
*Townsendia hookeri*	Asteraceae	yes	GA	A	yes	S, L, G	[45,46,47]
*Townsendia incana*	Asteraceae	yes	GA	A	yes	S, L, N	[45]
*Townsendia leptotes*	Asteraceae	yes	GA	A	yes	S, L, N, G	[45]
*Townsendia montana*	Asteraceae	yes	GA	A	yes	S, L, N	[45]
*Townsendia parryi*	Asteraceae	yes	GA	A	yes	S, L, N, G	[45]
*Townsendia rothrockii*	Asteraceae	yes	GA	A	yes	S, G	[45]
*Townsendia scapigera*	Asteraceae	yes	GA	A	unknown	no	[45]
*Townsendia spathulata*	Asteraceae	yes	GA	A	unknown	no	[45]
*Townsendia strigosa*	Asteraceae	yes	GA	A	unknown	S, N	[45]
*Handroanthus ochraceus*	Bignoniaceae	no	SA	P	yes	S	[48]
*Boechera collinsii*	Brassicaceae	no	GA	P	no	no	[49]
*Boechera crandallii*	Brassicaceae	yes	GA	P	no	S	[49]
*Boechera divaricarpa*	Brassicaceae	yes	GA	P	no	no	[49]
*Boechera fendleri*	Brassicaceae	yes	GA	P	no	no	[49]
*Boechera holboellii*	Brassicaceae	yes	GA	P	no	no	[50]
*Boechera lemmonii*	Brassicaceae	yes	GA	P	no	no	[49]
*Boechera lyallii*	Brassicaceae	yes	GA	P	no	no	[49]
*Boechera microphylla*	Brassicaceae	yes	GA	P	no	no	[49]
*Boechera pallidifolia*	Brassicaceae	yes	GA	P	no	no	[49]
*Boechera pauciflora*	Brassicaceae	yes	GA	P	no	no	[49]
*Boechera pendulocarpa*	Brassicaceae	yes	GA	P	no	no	[49]
*Boechera perennans*	Brassicaceae	no	GA	P	no	no	[49]
*Boechera pinetorum*	Brassicaceae	yes	GA	P	no	no	[49]
*Boechera puberula*	Brassicaceae	yes	GA	P	no	no	[49]
*Boechera retrofracta*	Brassicaceae	yes	GA	P	yes	S	[49]
*Boechera sparsiflora*	Brassicaceae	yes	GA	P	no	no	[49]
*Boechera spatifolia*	Brassicaceae	yes	GA	P	no	no	[16]
*Boechera stricta*	Brassicaceae	yes	GA	P	no	S	[49]
*Boechera williamsii*	Brassicaceae	yes	GA	P	no	no	[49]
*Hypericum maculatum*	Hypericaceae	yes	GA	A or P	yes	no	[51,52]
*Hypericum perforatum*	Hypericaceae	yes	GA	A or P	yes	S, L, N	[51,52]
*Eriotheca gracilipes/pubescens*	Malvaceae	no	SA	P	yes	S, L	[53]
*Nigritella nigra complex*	Orchidaceae	yes	SA	A	yes	S, L, N, G	[54,55]
*Spiranthes magnicamporum*	Orchidaceae	no	SA	A	unknown	S	[56]
*Spiranthes ochroleuca*	Orchidaceae	no	SA	A	unknown	S, N	[56]
*Limonium algarvense*	Plumbaginaceae	no	GA	A	yes	S	[57]
*Limonium binervosum/ovalifolium*	Plumbaginaceae	no	GA	A	yes	S, L, N, G	[57,58]
*Bouteloua curtipendula*	Poaceae	no	GA	P	yes	no	[2,59]
*Calamagrostis stricta*	Poaceae	yes	GA	A	yes	S	[60]
*Paspalum intermedium*	Poaceae	no	GA	P	yes	S, L, N	[61]
*Paspalum simplex*	Poaceae	no	GA	P	yes	S, L, N	[62]
*Poa cusickii* ssp. *cusickii*	Poaceae	yes	GA	A	yes	S	[63]
*Poa pringlei*	Poaceae	yes	GA	A	unknown	S, N	[63]
*Ranunculus auricomus* agg.	Ranunculaceae	yes	GA	P	yes	S, L, N, G	[64,65]
*Ranunculus kuepferi*	Ranunculaceae	yes	GA	P	yes	S, L, N, G	[66,67]
*Ranunculus parnassifolius* s.l.	Ranunculaceae	yes	GA	P	yes	S, L, N, G	[68]
*Amelanchier bartramiana*	Rosaceae	no	GA	P	yes	S, N	[69,70]
*Amelanchier cusickii/alnifolia*	Rosaceae	yes	GA	P	yes	S, N	[69,70]
*Crataegus ser. Douglasiana*	Rosaceae	no	GA	P	yes	S, L, N	[71,72]
*Potentilla crantzii*	Rosaceae	yes	GA	P	yes	S, L, N, G	[73]
*Potentilla puberula*	Rosaceae	yes	GA	P	yes	S, N	[74]
*Rubus* subg. *Rubus*	Rosaceae	no	GA	P	yes	S, L, N	[75,76]

* usually A and P are not completely exclusive, here the predominant mode is reported. ** meaning that a part of the distribution area is allopatric between sexuals and apomicts, but not completely exclusive. S = most general pattern, L, N, and G are more specific categories.

## Data Availability

Not applicable.

## Supplementary Materials

The following supporting information can be downloaded at: https://www.mdpi.com/article/10.3390/plants12040844/s1, Supplementary Table S1: Presence/absence Table of data reported in Table 1 and candidates of putative GP patterns with uncertain data; Supplementary Table S2: Associations between the main putative causal factors. References [2,12,28,122,123] are cited in the Supplementary Materials.
