# Supplementary material for: Geographical Parthenogenesis in Alpine and Arctic Plants
Source: Plants (Basel). 2023 Feb 13;12(4):844. doi: 10.3390/plants12040844 (PMC9959270; doi:10.3390/plants12040844)
Supplement: Supplementary file 1 [file plants-12-00844-s001.zip › plants-2200672-supplementary.pdf]

Supplementary Table S1. Candidates of putative GP patterns with uncertain data

Taxon	Family	Arctic/alpine	Type of apomixis	Type of pollination	Ploidy difference of apomicts/sexuals	GP pattern	Record
Leontopodium alpinum[1]	Asteraceae	yes	GA	A	yes	?	[1]
Draba spectabilis/spectrobrachia[2]	Brassicaceae	yes	?	?	?	?	[2]
Calamagrostis purpurascens[3]	Poaceae	yes	?	?	?	?	[2]
Poa alpina[3]	Poaceae	yes	GA, SA, vivipary	P	yes	?	[1, 2]
Poa fendleriana[4]	Poaceae	yes	GA	A	?	?	[2]
Alchemilla spp.[5]	Rosaceae	yes	GA	A	unknown	?	[1]
Potentilla delphinensis[3]	Rosaceae	yes	GA	A	unknown	?	[1]
Potentilla nivea[3]	Rosaceae	yes	GA	A	unknown	?	[1]

[1]Only sexual reproduction in the Alps found more recently [3, 4]

[2]Original record does not give evidence for apomixis

[3]Geographical istribution of sexual/apomictic taxa unknown or not documented in publications

[4]Recorded as abstract only, no other published data

[5]Many obligate apomictic, polyploid taxa in the European Alps, but sexual relatives unknown

1. Hörandl, E., Evolution and biogeography of alpine apomictic plants. *Taxon* **2011,** *60,* 390-402.
2. Bierzychudek, P., Patterns in plant parthenogenesis. *Experientia* **1985,** *41,* 1255-1264.
3. Hörandl, E.; Dobes, C.; Suda, J.; Vit, P.; Urfus, T.; Temsch, E.M., . . . Ladinig, U., Apomixis is not prevalent in subnival to nival plants of the European Alps. *Annals of Botany* **2011,** *108,* 381-390. 10.1093/aob/mcr142
4. Pegoraro, L.; Baker, E.C.; Aeschimann, D.; Balant, M.; Douzet, R.; Garnatje, T., . . . Pellicer, J., The correlation of phylogenetics, elevation and ploidy on the incidence of apomixis in Asteraceae in the European Alps. *Bot. J. Linnean Soc.* **2020,** *194,* 410-422. 10.1093/botlinnean/boaa058

Supplementary Table S2. Presence/absence Table of data reported in Table 1 and statistical tests of associations between factors.

Data of Table 1 (columns arctic/alpine, Type of pollination, ploidy difference, and GP patterns separately for S, L, N, G were coded as presence/absence according to the literature cited in Table 1; cases with missing data were excluded from the respective analysis. Percentages of presences are visualized in Figure 1. Associations between the main putative causal factors were calculated as contingency tables and tested with chi square and Fisher's exact test using Past vs. 4.03 [1], the null hypotheses is no association). Degree of freedom = 1 in all calculations. Significant associations marked in bold.

Presence/absence matrix

Species	arctic/alpine	Separation	Pollination type	Ploidy difference	Larger	Northern	Glaciated
Antennaria friesiana	1	1	1	1	1	1	0
Antennaria monocephala	1	1	1	1	1	1	0
Antennaria parlinii	0	1	1	0	0	0	1
Antennaria rosea complex	1	1	1	1	1	1	0
Arnica alpina	1	1	1	1	1	1	0

Arnica amplexicaulis	1	1	1	1	1	1	0
Arnica angustifolia	1	1	1	1	0	0	1
Arnica chamissonis	1	1	1	1	1	1	0
Arnica lessingii	1	1	1	1	1	1	0
Arnica lonchophylla	1	1	1	1	1	1	1
Arnica louiseana	1	1	1	1	1	1	0
Arnica mollis	1	1	1	1	1	1	0
Chondrilla juncea	0	1	1	1	1	1	0
Crepis acuminata	0	0	1	1	0	0	0
Crepis bakeri	1	1	1	1	1	1	0
Crepis exilis	1	1	1	1	1	1	0
Crepis modocensis	1	1	1	1	1	1	1
Crepis monticola	1	1	1	1	1	1	0
Crepis occidentalis	1	1	1	1	1	1	1
Crepis pleurocarpa	1	1	1	1	1	1	0
Erigeron strigosus	0	1	1	1	1	1	0
Eupatorium altissimum	0	1	1	1	1	1	1
Eupatorium cuneifolium	0	1	1	1	1	1	0
Eupatorium lecheaefolium	0	1	1	1	1	1	0
Eupatorium leucolepis	0	1	1	1	1	1	0
Eupatorium pilosum	0	1	1	1	1	1	0
Eupatorium rotundifolium	0	1	1	1	1	1	0
Eupatorium sessilifolium	0	1	1	1	1	1	1
Hieracium alpinum	1	1	1	1	1	1	1
Hieracium intybaceum	1	1	1	1	0	0	0
Hieracium pilosella s.l.	1	1	1	1	0	1	0
Parthenium argentatum	0	1	0	1	1	1	0
Taraxacum Sect. Alpestria	1	1	1	1	1	1	1
Taraxacum Sect. Ruderalia	1	1	1	1	1	1	0
Taraxacum Sect. Erythrosperma	1	1	1	1	1	1	0
Townsendia condensata	1	1	1	1	1	1	1
Townsendia exscapa	1	1	1	1	1	1	1
Townsendia grandiflora	1	1	1	1	0	0	0
Townsendia hookeri	1	1	1	1	1	0	1
Townsendia incana	1	1	1	1	1	1	0
Townsendia leptotes	1	1	1	1	1	1	1
Townsendia montana	1	1	1	1	1	1	0
Townsendia parryi	1	1	1	1	1	1	1
Townsendia rothrockii	1	1	1	1	0	0	1
Townsendia scapigera	1	0	1		0	0	0
Townsendia spathulata	1	0	1		0	0	0
Townsendia strigosa	1	1	1		0	1	0
Handroanthus ochraceus	0	1	0	1	0	0	0
Boechera collinsii	0	0	0	0	0	0	0
Boechera crandallii	1	1	0	0	0	0	0
Boechera divaricarpa	1	0	0	0	0	0	0
Boechera fendleri	1	0	0	0	0	0	0
Boechera holboellii	1	0	0	0	0	0	0

Boechera lemmonii	1	0	0	0	0	0	0
Boechera lyallii	1	0	0	0	0	0	0
Boechera microphylla	1	0	0	0	0	0	0
Boechera pallidifolia	1	0	0	0	0	0	0
Boechera pauciflora	1	0	0	0	0	0	0
Boechera pendulocarpa	1	0	0	0	0	0	0
Boechera perennans	0	0	0	0	0	0	0
Boechera pinetorum	1	0	0	0	0	0	0
Boechera puberula	1	0	0	0	0	0	0
Boechera retrofracta	1	1	0	1	0	0	0
Boechera sparsiflora	1	0	0	0	0	0	0
Boechera spatifolia	1	0	0	0	0	0	0
Boechera stricta	1	1	0	0	0	0	0
Boechera williamsii	1	0	0	0	0	0	0
Hypericum perforatum	1			1			
Hypericum maculatum	1			1			
Eriotheca gracilipes/pubescens	0	1	0	1	1	0	0
Nigritella nigra complex	1	1	1	1	1	1	1
Spirnathes magnicamporum	0	1	1		0	0	0
Spiranthes ochroleuca	0	1	1		0	1	0
Limonium algarvense	0	1	1	1	0	0	0
Limonium binervosum/ovalifolium	0	1	1	1	1	1	1
Bouteloua curtipendula	0	0	0	1	0	0	0
Calamagrostis stricta	1	1	1	1	0	0	0
Paspalum intermedium	0	1	0	1	1	1	0
Paspalum simplex	0	1	0	1	1	1	0
Poa cusickii ssp. *cusickii*	1	1	1	1	0	0	0
Poa pringlei	1	1	1		0	1	0
Ranunculus auricomus agg.	1	1	0	1	1	1	1
Ranunculus kuepferi	1	1	0	1	1	1	1
Ranunculus parnassifolius s.l.	1	1	0	1	1	1	1
Amelanchier bartramiana	0	1	0	1	0	1	0
Amelanchier cusickii/alnifolia	1	1	0	1	0	1	0
Crataegus ser. Douglasiana	0	1	0	1	1	1	0
Potentilla crantzii	1	1	0	1	1	1	1
Potentilla puberula	1	1	0	1	0	1	0
Rubus sect. *Rubus*	0	1	0	1	1	1	0
Sum of cases	**90**	**88**	**88**	**84**	**88**	**88**	**88**
Presence	64	68	54	65	48	53	21
Absence	26	20	34	19	40	35	67

Percentages of presences	71,1%	77,3%	61,4%	77,4%	54,5%	60,2%	23,9%

Test statistics

Pairwise comparisons	No. of cases	Chi-square p	Fisher's exact p	association
**Arctic/alpine - geographical separation (S)**	**90**	**0.396**	**0.498**	**yes**
Autonomous apomixis (A) - geographical separation (S)	88	0.022	0.033	no
**Autonomous apomixis (A) - larger distribution area of apomicts (L)**	**88**	**0.359**	**0.448**	**yes**
**Autonomous apomixis (A) - more polar distribution of apomitcs (N)**	**88**	**0.887**	**1.000**	**yes**
Autonomous apomixis (A) – only apomicts in previously glaciated areas (G)	88	4.90E-07	7.87E-07	no
Ploidy difference - Autonomous apomixis (A)	82	0.012	0.019	no
**Ploidy difference - geographical separation (S)**	**84**	**1.000**	**1.000**	**yes**
Ploidy difference - larger distribution area of apomicts (L)	84	0.008	0.013	no
Ploidy difference - more polar distribution of apomicts (N)	84	0.019	0.03	no
Ploidy difference – only apomicts in previously glaciated areas (G)	84	1.11E-11	1.09E-11	no

1. Hammer, O. Past 4 - the Past of the Future. (PAleontological Statistics). https://www.nhm.uio.no/english/research/resources/past/ (30th Nov 2022),
